# Electronic health records tools for treating obesity among adult patients in primary care: A scoping review

**DOI:** 10.1016/j.obpill.2025.100161

**Published:** 2025-01-19

**Authors:** Jyotsna Ghosh, Kimberly A. Gudzune, Jessica L. Schwartz

**Affiliations:** a Johns Hopkins University School of Medicine, Baltimore, MD, USA; b American Board of Obesity Medicine Foundation, Denver, CO, USA

**Keywords:** Obesity management, Clinical decision support systems, Primary health care, Review

## Abstract

**Background:**

Electronic health record (EHR)-based tools, such as clinical decision support systems (CDSS), support practitioners to promote evidence-based care, which may include obesity treatment. Our objective was to identify obesity-focused CDSS for adult patients in primary care settings to describe their designs, associated primary care practitioner (PCP) training, and outcomes among PCPs and patients.

**Methods:**

We conducted a scoping review to identify and map available evidence using a search strategy for citations in MEDLINE from February 2009 to June 2024. We extracted information from included studies that described EHR-based CDSS tools designed to support obesity care (e.g., clinical decision support, counseling) for adult patients in primary care settings. We mapped common tool features to support weight management and synthesized key lessons learned during implementation of these tools.

**Results:**

Of the 445 citations identified in our search, we included 13 citations reporting on 8 studies. The most common features across EHR-based CDSS tools were 1) identifying overweight or obesity using BMI (88 %) and 2) suggesting treatment strategies (88 %), particularly lifestyle modifications. Most studies provided limited information on the training PCPs received. Few PCPs used the CDSS with eligible patients (<20 %), describing these tools as cumbersome and lacking clinical workflow integration. Novel approaches included using CDSS during weight management-dedicated visits or for referral to obesity medicine physicians, which both showed promising early results of patients achieving weight reduction.

**Conclusion:**

There is a growing body of evidence for obesity-focused CDSS among adult patients in the primary care setting. Our review identified three key lessons that may inform future health system implementation: 1) EHR-based CDSS tools need to be easy to use and integrate with clinical workflows; 2) PCPs need training on these tools for obesity treatment; and 3) Primary care workflow or work-scope may need to be modified to address obesity.

## Graphical abstract

**Figure:**
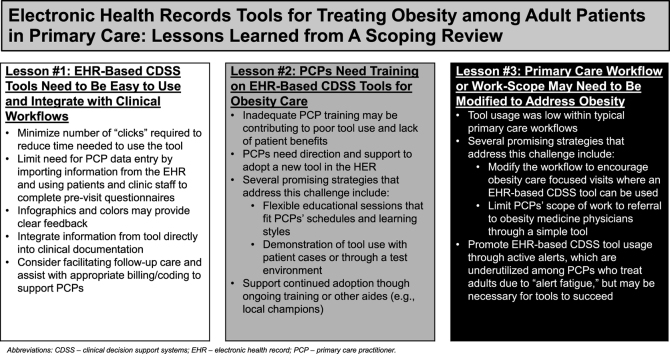

## 1 Introduction

Since 1996, the U.S. Preventive Task Force has recommended that clinicians screen adults for obesity and offer counseling to patients with this chronic disease [1]. Obesity contributes to multi-morbidity [2,3], but modest weight loss of 5 %–10 % improves quality of life and helps manage weight-related conditions (e.g., type 2 diabetes mellitus) [[4], [5], [6]]. Primary care is an important setting for clinicians to partner with patients on obesity treatment [7]. When primary care practitioners (PCPs) offer such counseling, research has found that the interaction supports patients' behavior change [8]. Furthermore, PCPs’ counseling has been associated with modest weight loss and favorable lifestyle changes [9,10].

Despite this benefit, most primary care visits for patients with obesity do not include weight management discussions [11]. Several challenges may contribute to this care gap. PCPs most often cite time constraints as a primary reason for the lack of obesity counseling [12], which is further compounded when balancing multiple competing priorities during brief primary care visits [13]. Inadequate reimbursement for obesity care also limits PCPs delivery of these services [12]. PCPs report being insufficiently trained to effectively and sensitively address obesity [12,14] – a chronic disease that is complex and may be an emotional subject for many individuals. Training PCPs in evidence-based techniques, such as the 5As framework (Ask, Advise, Assess, Assist, and Arrange), increases their self-efficacy for obesity counseling as well as their belief in the benefits of their counseling to patients [15,16]. People living with obesity who receive such counseling report greater progress toward weight goals and positively rate their care experience [17]. Finally, PCPs' weight bias may be a barrier to counseling, as patients frequently identify clinicians as sources of weight stigmatization and some PCPs may believe weight loss to be the patient's sole responsibility [18,19].

Clinical decision-support systems (10.13039/100018140CDSS) within the electronic health record (10.13039/100000081EHR) have the potential to effectively and efficiently support clinicians at the point-of-care. 10.13039/100018140CDSS are digital tools that have the potential to support practitioners at the point-of-care to promote evidence-based weight management guidelines [20]. With the 2009 passage of the Health Information Technology for Economic and Clinical Health (HITECH) Act, EHRs have become widely used given the legislation's financial incentives and potential penalties [21]. In a study examining all U.S. healthcare institutions, EHR use has grown from 7 % in 2009 to 81 % in 2019 [22], making EHR capabilities now accessible to most clinicians. Prior studies have successfully incorporated CDSS for chronic disease management into the EHR, including asthma, hypertension and diabetes [23]. EHR-based 10.13039/100018140CDSS might be leveraged to support obesity counseling in primary care settings [10]. A 2013 systematic review found that most EHR interventions focused on calculating body mass index (BMI) and few randomized controlled trials (RCTs) tested these tools [24]. In addition, most studies in this review focused on pediatric patients rather than adults with obesity.

Our objective was to identify 10.13039/100000081EHR interventions that supported obesity evaluation and treatment for adult patients in primary care settings. By conducting a scoping review, we aimed to describe the design of these EHR-based CDSS tools (e.g., features, components) as well as the training received by PCPs to use these tools. We also outlined the available evidence for these tools on both PCP and patient outcomes.

## 2 Methods

### 2.1 Design & data sources

We conducted a scoping review, which is a methodology to identify and map available evidence as well as detect knowledge gaps [25]. We created a search strategy to identify citations in MEDLINE from February 2009 to June 2024 (**Supplemental Materials 1**). Our search strategy aimed to identify EHR-based 10.13039/100018140CDSS tools designed to support evaluation and treatment of obesity in primary care settings. We reviewed the reference lists of each included article to identify additional citations and conducted an online search to identify outcomes from research protocols. We contacted authors of included studies requesting outcomes information if no published article was identified; however, no additional information was sent in response to these queries.

### 2.2 Study selection

The study team reviewed each article for eligibility against prespecified criteria. We included studies that described EHR-based 10.13039/100018140CDSS tools designed to support obesity care among PCPs (e.g., clinical decision support, counseling). We included studies within adult patient populations. We excluded studies that were limited to pediatric or special populations (e.g., cancer survivors, pregnancy), tools that focused on obesity care outside the clinic setting, tools that focused on obesity prevention or weight maintenance, tools limited only to diagnosis of overweight or obesity, and studies occurring outside of the United States or prior to the HITECH Act (February 2009) given the increase in EHR adoption since its enactment [22]. Using these criteria, the study team conducted a title review, followed by abstract review and then article review.

### 2.3 Data extraction

Study team members extracted data on study design, population, and setting. We also extracted information on the characteristics of each EHR-based CDSS tool, including function, features, and components. We defined a tool as an “active alert” if it used a pop-up window to alert the PCP. We noted whether the tool identified a diagnosis of overweight or obesity for the PCP using body mass index (BMI) or assisted the PCP in documenting a diagnosis of overweight or obesity in the medical record. We determined whether each tool had any of the following clinical decision support features: setting goals, recommending laboratory tests, suggesting treatment strategies, referrals (e.g., dietitian, weight management program), patient resources (e.g., educational handouts), facilitating order entry (e.g., additional testing, referrals), or facilitating follow-up care with the PCP. We also ascertained whether the tool assisted PCPs with billing/coding (e.g., diagnosis or visit codes). We extracted information about PCPs’ training to use the tool and other support resources provided to them. Finally, we extracted information about outcomes, when available, including implementation barriers and challenges with the EHR-based CDSS tool, changes in PCP clinical practice habits, as well as changes in patient outcomes (e.g., changes in behavior, weight loss).

### 2.4 Data synthesis

Using a qualitative approach, we mapped common features of EHR-based 10.13039/100018140CDSS tools to support obesity care. We synthesized key lessons learned during implementation of these tools.

## 3 Results

Overall, we identified 445 citations in our search, and we excluded 367 at title review, 29 at abstract review, and 36 at full-text review. We included 13 citations that reported on 8 studies among adult patients with overweight/obesity (**Supplemental Materials 2**).

### 3.1 EHR-based CDSS tool design

Table 1 summarizes the features and components of each EHR-based CDSS tool. The most common feature across all tools was identifying overweight or obesity using BMI (88 %). Some tools prompted the addition of height or weight at the visit to facilitate BMI calculation by the EHR, which had been identified by design teams as a barrier to diagnosis and care [29,33,34]. Less common features included documenting overweight/obesity in the medical record (e.g., problem list, visit note) (63 %) and supporting PCPs in setting specific goals with patients (e.g., weight reduction target, specific lifestyle changes) (50 %). Only two tools aided PCPs with billing and coding (25 %).

**Table 1:** Summary of components in electronic health record clinical decision support systems for obesity care among adult patients in primary care settings.

Study	Study Year(s)	Components of CDSS										
		Identify Overweight/Obesity^a^	Document Overweight/Obesity	Active Alert	Set Goals	CDSS Suggests …				Facilitate Order Entry	Facilitate PCP Follow-Up	Assist Billing/Coding
						Laboratory Tests	Treatment Strategies	Referrals	Resources			
Tang [26] b	2010	Yes	Yes	No	Yes	No	Yes	No	Yes	Yes	Yes	No
Baer [27,28]	2011–13	Yes	Yes	Yes	Yes	No	Yes	Yes	Yes	No	No	No
Steglitz [29]	2012–13	Yes	Yes	No	Yes	No	Yes	No	No	No	No	No
Gangadhar [30]	2014–15	Yes	No	Yes	No	No	Yes	Yes	No	No	Unclear	No
Fitzpatrick [31] b	2015–16	Yes	No	Yes	No	No	Yes	Yes	Yes	Yes	No	No
Vesely [32] b	2016	Yes	Yes	No	No	Yes	Yes	Yes	Yes	Yes	Yes	Yes
Perreault [[33], [34], [35], [36]] b	2018–19 2019–20 2020–25	Yes	Yes	Unclear	Yes	Yes	Yes	Yes	No	Yes	Yes	Yes
Griauzde [37,38] b	2020–21	No	No	No	No	Yes	No	Yes	No	Unclear	No	No

Abbreviations: CDSS – clinical decision support systems; PCP – primary care practitioner.

a Identified overweight or obesity using body mass index.

b Electronic health record tool created within Epic; all other studies used other electronic health record systems.

Three EHR-based 10.13039/100018140CDSS 10.13039/100000081EHR tools were active alerts (38 %) [27,28,30,31], where a pop-up notified the PCP when the patient's 10.13039/100000993BMI met a prespecified threshold and was accompanied by additional clinical decision support features (e.g., goal setting, placing orders). Another employed a strategy where the tool was only used during clinic visits dedicated to weight management [33,34], rather than attempting to integrate obesity treatment into a typical primary care visit. Patients completed a pre-visit questionnaire about their behaviors, goals, and past weight-loss attempts that was completed by medical assistants or electronically [[33], [34], [35], [36]]. These pre-visit questionnaires were intended to increase PCP efficiency during these visits. Another tool aimed to support PCPs in identifying adults with obesity and referring them for evaluation and treatment by an obesity medicine physician [37,38], rather than directly treating the obesity themselves given PCPs inadequate training and time.

All EHR-based CDSS tools were designed with the goal of increasing PCPs’ awareness and use of clinical practice guidelines for obesity treatment. Tools typically attempted to facilitate implementation of clinical practice guidelines through suggested treatment strategies (88 %) and referrals (75 %). All studies that suggested treatment strategies provided suggestions for lifestyle changes, while only three discussed anti-obesity medications and metabolic-bariatric surgery referral as treatment options [27,28,30,[33], [34], [35], [36]]. One study provided scripted counseling approaches to help communicate treatment approaches [31]. Half of the tools enabled PCPs to provide educational patient handouts, while only three tools facilitated follow-up with the PCP for weight management (38 %).

### 3.2 PCP training on EHR-based CDSS tool and other strategies to support use

Table 2 includes information on PCP training and support with each tool. Overall, most studies provided limited information on the training PCPs received on the tool – some studies included no information on this topic (38 %), while others provided no detail on length of training or its content (38 %). When reported, the training usually occurred only once, although one study provided an optional booster session [26]. Two studies also employed local champions to support tool use in addition to PCP training [26,35,36]. Interestingly, one study provided PCPs with continuing medical education credit to complete the training [35,36]. One study identified inadequate PCP education on how to use the tool as a major challenge in their study [26].

**Table 2:** Overview of study design and key outcomes for electronic health record clinical decision support systems for obesity care among adult patients in primary care settings.

Study	Study Design (Location) *Patient Population*	PCP Training and Support for CDSS Tool	Summary of Key PCP Outcomes	Summary of Key Patient Outcomes
Tang [26]	RCT (Illinois) *BMI 27-29.*9 kg/m^2^	• 1-h ​group training on tool (counseling template and order set) • Optional 20-min ​one-on-one hands-on 20 booster session • Tool use promoted by a local physician champion	• 30 PCPs (15 intervention, 15 control) • Tool used in only 11 % of alerts; however, when tool was used nearly all encounters had documented treatment plan and ICD diagnosis of overweight • Intervention PCPs significantly more likely to perform weight-related counseling and diagnose overweight during new or preventive visits than control PCPs • PCPs identified time as the biggest barrier to use (7.5 min to use), followed by too complexity and patient disinterest	• Interviews with 61 patients whose PCPs used the tool • Nearly all recalled discussing weight during their visit – 93 % recalled setting a weight management plan, 84 % made lifestyle changes in response, and 56 % reported losing weight
Baer [27,28]	Cluster-randomized RCT at clinic-level (Massachusetts) *BMI* ≥ 25 kg/m^2^	• Brief presentation at each intervention clinic delivered during a regularly scheduled practice meeting • Quick reference guide with information about the new EHR features	• 23 primary care clinics (11 intervention, 12 control) • Tool increased diagnosis of overweight/obesity on the problem list (35 % increase in intervention, 8 % decrease in control) • No significant differences in nutritional counseling or anti-obesity medication prescription between groups • Intervention PCPs reported increased confidence in weight-loss counseling compared to control; all PCPs wanted more help in creating obesity treatment plans • Intervention PCPs perceived the tool to be very cumbersome and disruptive to their workflow – desired fewer “clicks” to use the tool and fewer reminders	• 35,665 eligible patients had EHR values examined; 590 patients completed post-visit survey • No significant difference in mean weight change at 6 or 12 months between groups • More patients at intervention clinics recalled discussing weight loss with their PCP than control clinics (61 % vs 54 %)
Steglitz [29]	Prospective observational study with nested case-control (Illinois) BMI ≥30 kg/m^2^	• A single training session to introduce the EHR tool • Clinical protocol implemented to prompt MAs to measure weight and height; complete screening questionnaire	• 40 PCPs (30 % completed survey about tool) • Tool used with only 55 patients (2 % of adults with obesity) • Tool increased PCPs self-reported discussions on physical activity and diet, but did not increase creating an action plan to address obesity • PCPs reported that the tool improved their recognition of obesity, but they did not find it useful for improving other care processes • Tool did not increase PCPs' documentation of obesity on the problem list pre-post	• Nested case-control analysis found patients exposed to EHR tool were more likely to receive weight-loss counseling than controls, but there were no significant between-group differences in BMI change (n = 92) • Most frequent behavioral goals set by patients were “eating more healthy foods” and “increasing physical activity”
Gangadhar [30]	Cluster-randomized RCT at clinic-level (Multiple US states) *BMI* ≥ 25 kg/m^2^*(age 18–64)* *BM*I ≥ 30 kg/m^2^*(age* ≥*65)*	*Not described*	• 5871 primary care practices (4987 intervention, 881 control) • 10 % of alerts prompted follow-up plan documentation	• Patients with BMI recorded twice during study period (sample size NR) • No significant difference in mean weight change among patients with overweight/obesity between groups
Fitzpatrick [31]	Cluster-randomized RCT at clinic-level (Illinois) *BMI* ≥ 30 kg/m^2^	• IT personnel provided training on use and features of the tool • Leadership sent emails to PCPs describing the tool before implementation	• 14 primary care clinics (7 intervention, 7 usual care best practice alert with nutrition handouts) • Tool increased documentation of obesity treatment by 14 % (from 17 % to 31 %) in the intervention arm; however, no significant difference with comparator • Intervention PCPs referred only 2 % of patients for treatment • Intervention PCPs desired more education about how the tool worked as well as information about treatment referral options – desired fewer “clicks” to use the tool and better workflow integration, some liked that the alert changed from yellow to green when completed	• Surveys of 500 patients at intervention clinics • 60 % recalled discussing weight with PCP; 22 % received a nutrition handout, 17 % received a treatment referral, and 5 % attended an appointment with the referral service
Vesely [32]	Descriptive (Minnesota) *BMI ≥* 25 kg/m^2^	*Not described*	*None reported*	*None reported*
Perreault [33,34] [35,36]	Prospective evaluations of intervention compared to matched control (Colorado) *BMI* ≥ 25 kg/m^2^	• No training provided • Clinic workflow modified to have dedicated visits for weight management where EHR tool used •Clinical protocol implemented to prompt MAs to complete screening questionnaire	• 4 physicians in first pilot study (2 intervention, 2 control); 17 PCPs in second pilot study (10 intervention, 7 control) • Intervention increased the number of weight-focused visits as compared to usual care (16 % of eligible patients had a weight-focused visit in intervention clinic vs 8 % in usual care clinic) • Anti-obesity medication prescribed in 80 % of patients among intervention PCPs compared to 21 % of control	• Intervention patients had significantly greater weight loss than controls (7.3 % vs 2.3 %) in first pilot study (n = 417); however, there was no statistically significant difference in weight loss between groups in the second pilot study (n = 581)
	Stepped wedge RCT with randomization at clinic-level (Colorado & Wyoming) *BMI ≥* 25 kg/m^2^	• Online e-learning module on evidence-based weight management treatment (PCPs receive 2 CME credits) • Practice Champion at each site to engage leadership and support PCPs • Optional consultation support for PCPs built into EHR system • Patient completes questionnaires in advance of weight-focused visit to increase PCP efficiency	*None reported (trial ongoing)*	*None reported (trial ongoing)* 20,383 eligible patients included
Griauzde [37,38]	Retrospective evaluation of intervention compared to matched control cohort (Michigan) *BMI* ≥ 30 kg/m^2^ *+ ≥1 weight-related condition*	*Not described*	• PCPs referred 19 % of eligible patients to the weight navigation program	• 264 patients included (132 intervention, 132 matched control) • Referred patients achieved significantly greater weight loss than controls

Abbreviations: CDSS – clinical decision support systems; CME – continuing medical education; EHR – electronic health record; IT – information technology; MA – medical assistant; NR – not reported; PCP – primary care practitioner; RCT – randomized controlled trial.

### 3.3 PCP outcomes

Overall, seven studies reported outcomes among PCPs (Table 2). Studies often used interviews or surveys after study completion to determine these results. PCPs in several studies noted that a common challenge was that the EHR-based CDSS tool was cumbersome/complex and did not integrate into their clinical workflow [[26], [27], [28],^31^]. Time required to complete the tool was also noted as a challenge. One study found that PCPs needed 7.5 min for this activity, which they perceived as taking too much time [26]. PCPs’ actual use of the tool with eligible patients was low. For example, less than 20 % of alerts prompted physician action to treat obesity among adult patients [26,30,31,33,34,37,38].

### 3.4 Patient outcomes

We identified seven studies that reported outcomes among patients that received care from PCPs with access to the EHR-based CDSS tools (Table 2). Notably, one intervention was tested in two pilot studies and is currently being studied in a large clinical trial (ending in 2025) [35,36]. To determine patient outcomes, studies often used interviews or surveys after study completion as well as through data extraction from the EHR. When assessed, patients recalled discussing their weight with their PCP [[26], [27], [28],^31^].

Three studies found no significant difference in weight change at follow-up between intervention and comparator groups [[27], [28], [29], [30]]. The study that employed the tool during weight management focused visits showed significantly greater weight loss among patients as compared to controls in their first pilot study [33]; however, no weight loss difference was seen in the second pilot study [34]. This first pilot involved both PCPs and endocrinologists, whereas the second pilot included only PCPs. The study that aimed to refer patients to care with an obesity medicine physician also found that referred patients achieved significantly greater weight loss than controls [37,38]. Patient engagement with obesity treatment services was associated with weight loss [26,33,37,38], although engagement with these services in the studies was low.

### 3.5 Identified key lessons

Our review identified three key lessons: 1) EHR-based CDSS tools need to be easy to use and integrate with clinical workflows; 2) PCPs need training on these tools for obesity care; and 3) Primary care workflow or work-scope may need to be modified to address obesity. We provide additional information about these lessons learned in Table 3.

**Table 3:** Lessons learned regarding electronic health record clinical decision support systems for obesity care among adult patients in primary care settings.

**EHR-Based CDSS Tools Need to Be Easy to Use and Integrate with Clinical Workflows** • Minimize number of “clicks” required to reduce time needed to use the tool • Limit need for PCP data entry by importing information from the EHR and using patients and clinic staff to complete pre-visit questionnaires • Infographics and colors may provide clear feedback • Integrate information from tool directly into clinical documentation • Consider facilitating follow-up care and assist with appropriate billing/coding to support PCPs
**PCPs Need Training on EHR-Based CDSS Tools for Obesity Care** • Inadequate PCP training may be contributing to poor tool use and lack of patient benefits • PCPs need direction and support to adopt a new tool in the EHR • Several promising strategies that address this challenge include: o Flexible educational sessions that fit PCPs' schedules and learning styles o Demonstration of tool use with patient cases or through a test environment o Support continued adoption though ongoing training or other aides (e.g., local champions)
**Primary Care Workflow or Work-Scope May Need to Be Modified to Address Obesity** • Tool usage was low within typical primary care workflows • Several promising strategies that address this challenge include: o Modify the workflow to encourage obesity care focused visits where an EHR-based CDSS tool can be used o Limit PCPs' scope of work to referral to obesity medicine physicians through a simple tool o Promote EHR-based CDSS tool usage through active alerts, which are underutilized among PCPs who treat adults due to “alert fatigue,” but may be necessary for tools to succeed

Abbreviations: CDSS – clinical decision support systems; EHR – electronic health record; PCP – primary care practitioner.

## 4 Discussion

Our scoping review focused on describing EHR-based 10.13039/100018140CDSS tools that aimed to support evaluation and treatment of obesity. Overall, we identified eight studies describing 10.13039/100018140CDSS to support PCPs in evaluating and treating obesity among adult patients since the 2009 HITECH Act. These tools predominantly focused on aiding PCPs in identifying adult patients with overweight/obesity by using BMI as well as providing PCPs with suggested treatment approaches for these patients, particularly lifestyle modifications. Few tools supported PCPs in prescribing anti-obesity medication or discussing metabolic-bariatric surgery, which are both evidence-based treatment strategies typically associated with greater weight reduction than lifestyle alone [39,40]. Population-level studies have shown that few adults with obesity use anti-obesity medications or metabolic-bariatric surgery [41]. These findings suggest a need for clinical strategies to increase recommendation of these treatments. 10.13039/100018696Health systems in which development and implementation of obesity-focused 10.13039/100018140CDSS is being considered may well benefit from designing tools that support comprehensive, evidence-based treatment options for obesity among adults [42].

We found that PCPs' usage of EHR-based CDSS was low in most studies. When tools were used, however, patients had benefits that included losing weight, making lifestyle modifications, and being prescribed anti-obesity medications. Given such benefits, understanding the barriers to PCP use of these tools is of critical importance. PCP training on these obesity-focused CDSS appeared limited, and inadequate training may have negatively impacted PCPs’ tool use. In fact, one study specifically identified inadequate PCP education on the tool as a major challenge in their study [26]. For obesity-focused CDSS, PCPs may benefit from training on use of the CDSS itself as well as education on obesity and obesity treatment generally. Education on obesity care may be important given the well documented knowledge gaps in this area for clinicians [43]. A qualitative study among pediatric PCPs found that they preferred a combination of in-person and online training, and that these trainings would ideally be concise, interactive, and case-based for obesity-focused CDSS [44]. These PCPs also indicated that training should be ongoing and provide feedback to be most beneficial. Future research on obesity-focused CDSS should ensure adequate attention is given to PCP training on any tool implemented. This lesson is also important to health systems who may be considering implementing CDSS for obesity within their clinical practices.

The EHR-based CDSS used in these studies may have failed to satisfy PCP needs at the point-of-care – a weakness that may have contributed to low usage. Studies reported that using the tools took too long [26], required too many “clicks” [27,28,31], and disrupted clinician workflow [27,28,31]. Other research has found that patients are less actively participating in their care when physicians are physically engaged with the computer (e.g., keyboard activity, using the mouse) [45]. In addition, PCPs frequently report that they lack time for counseling during visits and must balance multiple competing health priorities [12,13]. Considering all these findings, PCPs’ clinical workflows may be essential to the success of CDSS for obesity care. Research suggests that the addition of medical scribes decreases physician EHR documentation burden and improves work efficiency and interactions with patients [46]. Therefore, the inclusion of scribes may be key when launching a CDSS. Other research has found that outcomes differ for active and passive alerts in the EHR. As compared to the passive alert, PCPs exposed to the active alert responded more often to the alert (60 % vs 30–41 %) and were more likely to use the associated note template (58 % vs 16–31 %) [44]. In this review, one of the studies that used active alerts found that the tool increased the frequency with which obesity was included as a diagnosis on the problem list [27,28]. Employing an active alert strategy may therefore be useful when a CDSS is used during typical primary care visits. Ultimately, future research and health system interventions should engage PCPs in the design and implementation of CDSS to ensure that the tool integrates into the primary care workflow. Human factors modeling methods may be a promising strategy to improve workflow through enhanced EHR functionality [47].

Rather than trying to incorporate obesity treatment into usual primary care workflows, two studies aimed to modify the primary care workflow or work-scope. One CDSS was implemented in the setting of weight management-dedicated primary care visits [[33], [34], [35], [36]], which were visits with the PCP scheduled to specifically discuss obesity treatment. In one pilot study, this strategy was associated with a greater number of weight management visits with PCPs as compared to control [33]. In addition, this study found greater use of anti-obesity medications and greater magnitude of weight reduction in the intervention arm as compared to control. This combination of CDSS with workflow modification is currently being tested in a large-scale stepped wedge RCT [35,36], which will test both implementation and patient outcomes. Another study modified PCP work-scope as the CDSS focused on aiding identification of adults with obesity and then referring them to an obesity medicine physician certified by the American Board of Obesity Medicine (ABOM) [37,38]. Prior research found that PCPs want support from peers trained in obesity medicine to improve obesity care [48]. ABOM-certified obesity medicine physicians offer services concordant with obesity treatment guidelines, including anti-obesity medications and perioperative metabolic and bariatric surgical care [49]. In this CDSS study, patients referred to obesity medicine achieved significantly greater weight reduction than controls [38]. We conclude that modification of the primary care workflow or work-scope may be promising strategies to improve outcomes for both PCPs and their patients.

In comparison to prior reviews, we identified several completed or ongoing RCTs testing the efficacy of EHR-based CDSS tools on PCP and patient outcomes (63 % of studies). A 2013 systematic review noted that only 27 % of included studies were RCTs, which included both adult and pediatric populations [24]. A 2017 systematic review identified only a single RCT testing a CDSS for obesity management among adult patients [10]. Overall, the evidence base on the impact of CDSS for adult weight management in primary care appears to be growing. Additional studies are needed to clarify best practices for implementation, particularly related to training and primary care workflow integration. Future research is also needed to determine CDSS impact on patient outcomes and how patient factors may alter outcomes.

## 5 Limitations

This review has several limitations. We conducted a scoping review to identify and map available evidence [25]. As we did not conduct a systematic review, we may not have identified all eligible studies relevant to EHR-based CDSS tools to treat obesity among adult patients in primary care settings. In addition, our review is subject to selection bias as we may not have identified all published studies or research protocols as we only searched a single database (MEDLINE) for published studies and we did not search grey literature (e.g., clinicaltrials.gov) for research protocols. We identified only eight studies for inclusion, and not all included PCP or patient outcomes. Given the limited assessment of patient outcomes in the included studies, we were unable to explore patient-level obstacles to these tools. Future research should prioritize the inclusion of patient perspectives regarding these tools as well as patient outcomes, as patients are equally critical to intervention success. Overall, the evidence base for these tools for adult obesity appears modest, which limits the generalizability of our findings. The lack of long-term patient outcomes in these studies weakens the case for broad adoption of such tools at this time. We did not evaluate the study quality or assess the risk of bias for any studies included.

## 6 Conclusions

There is a growing body of evidence regarding the effectiveness of EHR-based CDSS tools to evaluate and treat obesity among adults in the primary care setting. Our review identified three key lessons: 1) EHR-based CDSS tools need to be easy to use and integrate with clinical workflows; 2) PCPs need training on EHR-based CDSS tools for obesity care; and 3) Primary care workflow or work-scope may need to be modified to optimize clinical attention to obesity. These lessons may be particularly relevant to health systems that may be considering how to design and implement a CDSS for adult obesity treatment.

## Summary takeaway messages

• Most CDSS predominantly focus on identifying adults with overweight/obesity via BMI and providing PCPs suggested lifestyle modifications for these patients
• PCPs' CDSS usage was low in most studies, although when the tool was used adults living with obesity had benefits
• PCPs need training on obesity-focused CDSS

## Author contributions

JG, KAG, and JLS conceptualized this work, and all were involved with methodology and data extraction. JG wrote the first draft. All authors reviewed, edited, and approved the final submission and publication.

## Disclosures

JG declares that she has no competing interests. Johns Hopkins received grant funding from 10.13039/501100004191Novo Nordisk, which supported JLS and KAG in conducting the 2022 scoping review and a CDSS pilot study. During conduct of this work, KAG previously served as faculty at the Johns Hopkins School of Medicine and Medical Director for the American Board of Obesity Medicine. She is currently employed by the American Board of Obesity Medicine Foundation as Chief Medical Officer. She has received personal fees from Johns Hopkins ACG System and PRI-MED; personal fees for participation on advisory boards for Eli Lilly and Company and Novo Nordisk; and travel support from Eli Lilly and Company and Novo Nordisk. JLS has received grant funding from the Ardmore Institute for research outside the context of this work.

## Ethical adherence

This work only involved review of published or publicly available reports, and therefore, would not be considered human subjects research per U.S. Department of Health & Human Services definitions.

## Declaration of artificial intelligence (AI) and AI-assisted technologies

During the preparation of this work, the authors did not use AI.

## Source of funding

10.13039/501100004191Novo Nordisk funded a 2022 scoping review on behavioral and clinical decision support interventions for obesity in primary care, which was used to inform the design of a CDSS for obesity treatment in primary care. In 2024, the current author team updated and refined the prior review to the current form presented in this manuscript. The authors received no funding for this update.
