# Supplementary material for: Electronic health records tools for treating obesity among adult patients in primary care: A scoping review
Source: Obes Pillars. 2025 Jan 19;13:100161. doi: 10.1016/j.obpill.2025.100161 (PMC11795129; doi:10.1016/j.obpill.2025.100161)
Supplement: Multimedia component 1 [file mmc1.docx]

**Supplemental Materials for “*Electronic Health Records Tools for Treating Obesity among Adult Patients in Primary Care: A Scoping Review”***

**Table of Contents**

|  |  | **Page** |
| --- | --- | --- |
| Supplemental Materials 1. | Search Strategy for Scoping Review | 2 |
| Supplemental Materials 2. | Roster of Included Articles by Study | 3 |
|  |  |  |

**Supplemental Materials 1. Search Strategy for Scoping Review**

MEDLINE Search Strategy

"Obesity Management"[Mesh] OR "Weight Reduction Programs"[Mesh] OR "Overweight"[Mesh] OR "obesity management"[Title/Abstract] OR "weight reduction"[Title/Abstract] OR "weight loss"[Title/Abstract] OR "weight management"[Title/Abstract]

AND

"Electronic Health Records"[Mesh] OR "electronic health record*"[Title/Abstract] OR "EHR"[Title/Abstract] OR "electronic medical record*"[Title/Abstract] OR "EMR"[Title/Abstract]

AND

"Primary Health Care"[Mesh] OR "Physicians, Primary Care"[Mesh] OR "Primary Health Care"[Title/Abstract] OR "Primary Care"[Title/Abstract] OR "Primary Healthcare"[Title/Abstract]

AND

"2009/01/01"[Date - Publication] : "2024/06/30"[Date - Publication]

**Supplemental Materials 2. Roster of Included Articles, in Alphabetical Order by Study First-Author**

| **Study** | **Included Articles** |
| --- | --- |
| Baer | 1. Baer HJ, Wee CC, DeVito K, Orav EJ, Frolkis JP, Williams DH, et al. Design of a cluster-randomized trial of electronic health record-based tools to address overweight and obesity in primary care. *Clin Trials*. 2015; 12(4):374-83. PMID: 25810449. 2. H. Use of Electronic Health Records for Addressing Overweight and Obesity in Primary Care - Final Report. Rockville, MD: Agency for Healthcare Research and Quality, 2015. Available at: <https://digital.ahrq.gov/sites/default/files/docs/publication/k01hs019789-baer-final-report-2015.pdf>. Accessed on November 1, 2024. |
| Fitzpatrick | 1. Fitzpatrick SL, Dickins K, Avery E, Ventrelle J, Shultz A, Kishen E, et al. Effect of an obesity best practice alert on physician documentation and referral practices. *Transl Behav Med*. 2017;7(4):881-90. PMID: 28653221. |
| Gangadhar | 1. Gangadhar S, Nguyen N, Pesuit JW, Bogdanov AN, Kallenbach L, Ken J, et al. Effectiveness of a Cloud-Based EHR Clinical Decision Support Program for Body Mass Index (BMI) Screening and Follow-up. *AMIA Annu Symp Proc*. 2018; 2017:742-9. PMID: 29854140. |
| Griauzde | 1. Griauzde DH, Othman A, Dallas C, Oshman L, Gabison J, Markel DS, et al. Developing weight navigation program to support personalized and effective obesity management in primary care settings: protocol for a quality improvement program with an embedded single-arm pilot study. *Prim Health Care Res Dev*. 2022; 23:e14. PMID: 35234116. 2. Griauzde DH, Turner CD, Othman A, Oshman L, Gabison J, Arizaca-Dileo PK, et al. A Primary Care-Based Weight Navigation Program. *JAMA Netw Open*. 2024; 7(5):e2412192. PMID: 38771575. |
| Perreault | 1. Perreault L, Hockett CW, Holmstrom H, Tolle L, Kramer ES, Holtrop JS. PATHWEIGH Tool for Chronic Weight Management Built into EPIC Electronic Medical Record: Methods, Pilot Results and Future Directions. *J Obes Chronic Dis*. 2020; 4(1):42-8. <https://doi.org/10.17756/jocd.2020-036>. 2. Wild J, Kaizer A, Willems E, Kramer ES, Perreault L. Prelude to PATHWEIGH: pragmatic weight management in primary care. *Fam Pract*. 2023; 40(2):322-9. PMID: 35997768. 3. Suresh K, Holtrop JS, Dickinson LM, Willems E, Smith PC, Gritz RM, et al. PATHWEIGH, pragmatic weight management in adult patients in primary care in Colorado, USA: study protocol for a stepped wedge cluster randomized trial. *Trials*. 2022; 23(1):26. PMID: 35012628. 4. Perreault L, Suresh K, Rodriguez C, Dickinson LM, Willems E, Smith PC, et al. Baseline Characteristics of PATHWEIGH: A Stepped-Wedge Cluster Randomized Study for Weight Management in Primary Care. *Ann Fam Med*. 2023; 21(3):249-55. PMID: 37217322. |
| Steglitz | 1. Steglitz J, Edberg D, Sommers M, Talen MR, Thornton LK, Spring B. Evaluation of an electronic health record-supported obesity management protocol implemented in a community health center: a cautionary note. *J Am Med Inform Assoc*. 2015;22(4):755-63. PMID: 25665700. |
| Tang | 1. Tang JW, Kusher RF, Cameron KA, Hicks B, Cooper AJ, Baker DW. Electronic tools to assist with identification and counseling for overweight patients: a randomized controlled trial. *J Gen Intern Med*. 2012; 27(8):933-9. PMID: 22402982. |
| Vesely | 1. Vesely JM, Pronk NP, Kottke TE, Marshall PS. Obesity Treatment at HealthPartners: Adaptation of Clinical Guidelines into Systems for Practice Operations. *Curr Obes Rep*. 2016;5(3):312-9. PMID: 27342444. |
